# Non-inferiority designs in clinical trials for antithrombotic therapy in TAVR patients: did we go too far away by cutting corners?

**DOI:** 10.3389/fcvm.2024.1327904

**Published:** 2024-01-18

**Authors:** Giulia Lorenzoni, Dario Gregori, Giuseppe Tarantini

**Affiliations:** ^1^ Unit of Biostatistics, Epidemiology and Public Health, Department of Cardiac, Thoracic, Vascular Sciences and Public Health, University of Padova, Padova, Italy; ^2^ Interventional Cardiology Unit, Department of Cardiac, Thoracic, Vascular Sciences and Public Health, University of Padova, Padova, Italy

**Keywords:** trial, non-inferiority, TAVR, non-inferiority margin, study design

## Introduction

Non-inferiority (NI) studies are designed to demonstrate that a new intervention is no worse than an existing one by a predetermined margin. This approach is instrumental when it is assumed that the new treatment would have certain advantages over the standard one without compromising efficacy. The clinical definition of this margin of acceptability in the potential loss of efficacy is critical: setting a margin that is too large may result in adopting a therapy that is, in fact, worse than the standard. Because of this and several other issues, NI has been severely criticized and often discouraged (1).

Nevertheless, cardiovascular research continues to use NI designs. Their use in studies concerning antithrombotic therapy for transcatheter aortic valve replacement (TAVR) has expanded recently. In patients without an indication for oral anticoagulation (OAC), the use of dual antiplatelet therapy (DAPT) vs. single antiplatelet therapy (SAPT) is a matter of concern. Not least, in patients with an indication for OAC, the administration of OAC alone vs. dual therapy (OAC plus antiplatelet) is debated. Identifying the optimal therapy to balance the risk of thromboembolic events and bleeding in these patients remains challenging.

This study aimed to assess NI margins utilized in clinical trials on antithrombotic therapy for patients undergoing TAVR to quantify the additional risk permitted by these margins in the design of the studies.

## Methods

A systematic review was performed to identify NI randomized clinical trials on antithrombotic therapy in TAVI patients. The study was conducted according to the Preferred Reporting Items for Systematic Reviews and Meta-analyses (PRISMA) guidelines (2).

### Information sources and search strategy

The bibliographic search included PubMed, Embase, and CENTRAL (Cochrane Trial Registry). Embase and CENTRAL were searched via Ovid. No limits were applied to language and publication dates. The search string is reported in Supplementary Table S1.

### Eligibility criteria and selection process

Randomized clinical trials comparing antithrombotic therapies in TAVR patients employing an NI approach to test primary or secondary endpoints were eligible for inclusion. Published trial protocols and papers presenting trial results were also eligible to be included in the review. If more than one result was available for the same trial, the trial protocol was included in the review.

The selection process was done using the COVIDENCE software (3).

Conference proceedings, book chapters, trial registrations, systematic reviews, and meta-analyses were excluded, but they were checked for eligible papers.

### Data extraction and analysis

Characteristics of the included studies were extracted, including study design, interventions, and primary and secondary endpoints. Furthermore, data on assumptions made for sample size/power calculation for the endpoint tested for NI were extracted, including the event rate for the test drug, event rate for the standard drug, and NI margin. The NI was reported as an absolute risk difference (ARD). If the NI margin was reported as the hazard ratio, the following equation (4) was employed to derive the event rate in the intervention group admitted by the NI margin:

$$\mathrm{HR}=\frac{\log(1-p_{t})}{\log(1-p_{s})}$$

where $p_{t}$ is the outcome probability in the intervention group and $p_{s}$ is the outcome probability in the standard group.

The absolute NI margin was then calculated as ARD, i.e., the difference between the event rate in the intervention group admitted by the NI margin and the expected event rate in the standard group. Finally, the number needed to harm (NNH) was calculated as the reciprocal of the NI.

Information was extracted from the published study protocols or methods section of published trial results. If any amendments were made in the analysis stage, they were not considered in the study because the review aimed at evaluating NI margin assumptions employed in the design stage.

The data extraction tool was based on an Excel file.

## Results

The search retrieved 4,206 records (Supplementary Figure S1 for the PRISMA flowchart). After duplicate removal, 3,348 records underwent title/abstract screening. Finally, 72 records underwent full-text screening.

The review included three trials (Table 1) (5–7). They were published between 2016 and 2018. All the studies planned to test for NI a composite endpoint, a primary efficacy one in the ENVISAGE-TAVI AF and the GALILEO, and a secondary net-clinical benefit endpoint in the POPular TAVI. The ENVISAGE-TAVI AF planned a hierarchical testing strategy that involved testing for NI also a primary safety endpoint if the NI of the primary efficacy endpoint was met. The GALILEO study employed a hierarchical testing strategy too: the superiority test of the primary efficacy endpoint was hierarchically preceded by a test for NI. The sample size for the GALILEO study was determined for the superiority testing.

**Table 1:** Characteristics of the included studies.

Trial	Year of protocol publication	Intervention	Comparator	Endpoints evaluated for NI	Sample size	Expected event rate (comparator group)	Expected event rate (intervention group)	NI margin	NNH
ENVISAGE-TAVI AF (NCT02943785)	2018	Edoxaban	Vitamin K antagonist as approved in countries	**Hierarchical testing strategy**: Primary efficacy endpoint planned to be tested for NI. If proven, primary safety endpoint planned to be tested for NI too. **Primary efficacy endpoint**: NACE (all-cause death, myocardial infarction, ischemic stroke, systemic embolic events, valve thrombosis, or major bleeding) **Primary safety endpoint**: ISTH-defined major bleeding	1,400	14%	13.3%	4.8%	21
POPular TAVI (cohort A) (NCT02247128)	2016	Aspirin	Aspirin + clopidogrel	**Secondary net-clinical benefit endpoint** Composite of cardiovascular death, non–procedural-related bleeding, myocardial infarction, or stroke	684	39%	34%	7.5%	14
POPular TAVI (cohort B) (NCT02247128)	2016	OAC	OAC + clopidogrel	**Secondary net-clinical benefit endpoint** Composite of cardiovascular death, non–procedural-related bleeding, myocardial infarction, or stroke	316	39%	31%	7.5%	14
GALILEO (NCT02556203)	2017	Rivaroxaban + acetylsalicylic acid for 90 days and then rivaroxaban alone	Clopidogrel + acetylsalicylic acid for 90 days and then acetylsalicylic acid alone	**Hierarchical testing strategy**: Testing of the superiority of the primary efficacy endpoint hierarchically preceded by a test for NI **Primary efficacy endpoint** Composite of all-cause death, any stroke, myocardial infarction, symptomatic valve thrombosis, pulmonary embolism, deep venous thrombosis, and non-central nervous system systemic embolism	1,520 (sample size determination based on superiority testing)	33%	26.4%	5.2%	20

NACE, net adverse clinical event; ISTH, International Society on Thrombosis and Hemostasis; NI, non-inferiority; OAC, oral anticoagulation.

The POPular TAVI included two cohorts of patients, one without a long-term indication for oral anticoagulant (cohort A) and the other with a long-term indication for oral anticoagulant (cohort B). However, the NI margin and the expected event rate in the standard group were the same for both cohorts. The POPular TAVI was the only study included in the review that reported the NI margin as ARD and the expected event rate in both the intervention and standard groups. The ENVISAGE-TAVI AF and the GALILEO studies reported the NI margin as HRs [for what concerns the GALILEO study, information about the NI was derived from the protocol published within the main trial publication (8)], so the event rate admitted by the NI margin for the treatment group was derived using the appropriate formula (4).

**Figure 1:**
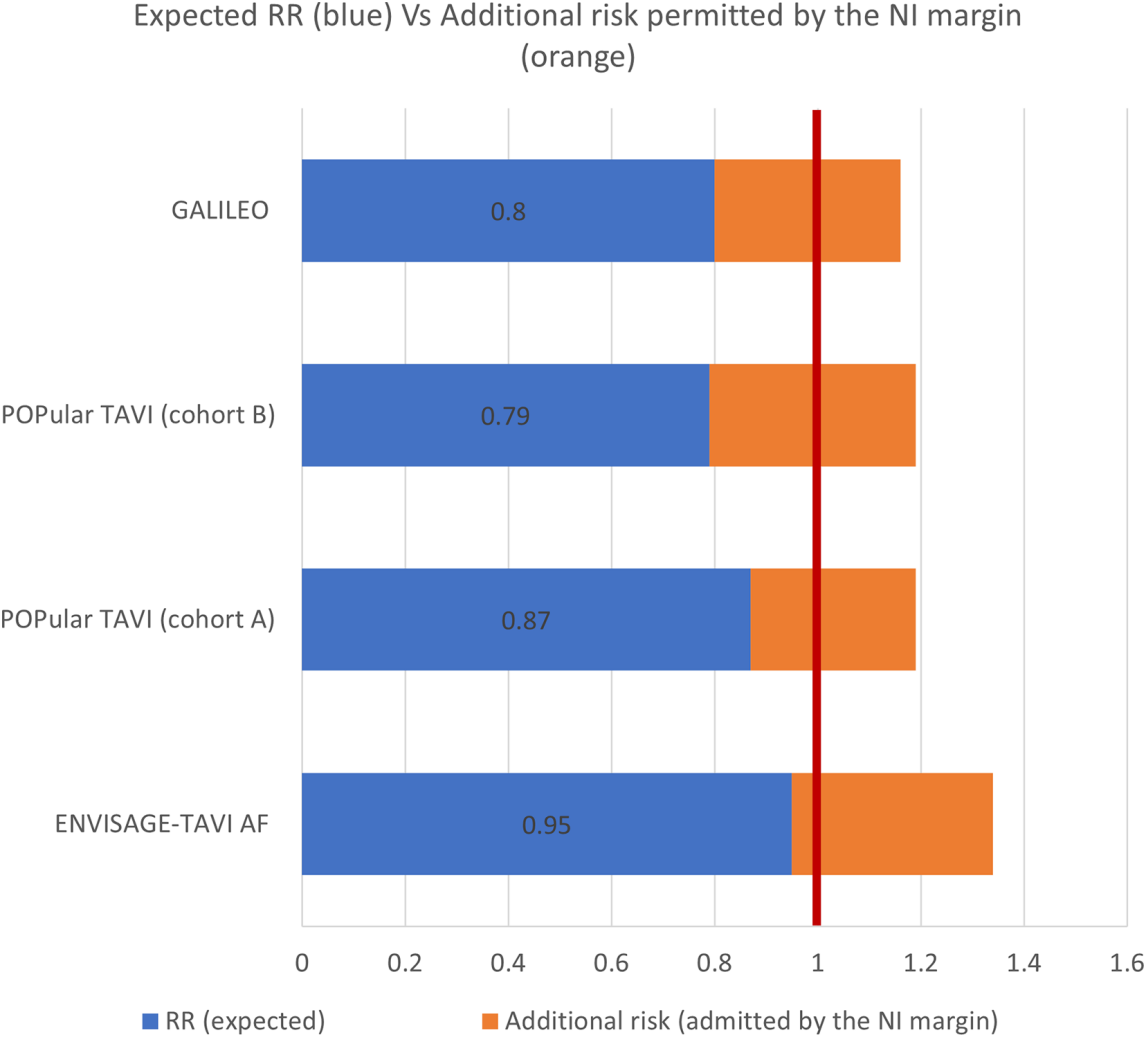
The figure presents, for each study, the expected effect of the intervention over the standard treatment expressed as relative risk (RR) (blue bar) and the excess of risk admitted by the NI margin (orange bar), resulting in a RR of 1.34 for the ENVISAGE-TAVI AF, 1.16 for the GALILEO, and 1.19 for the POPular TAVI. The vertical red line corresponds to no difference between the treatment and the comparator (RR equals to 1).

All studies justified the choice of the NI margin based on clinical appropriateness and literature review. The reported NI margin ranged from 4.8% to 7.5%, i.e., the studies considered a plausible and acceptable scenario that the test drug would result in an event rate up to 7.5 percentage points higher than the standard (Figure 1).

Based on such figures, NNH ranged from 14 (POPular TAVI) to 21 (ENVISAGE-TAVI AF). As an example, an NNH of 14 means that, for every 14 patients with no indication for OAC treated with SAPT instead of DAPT after TAVR, 1 additional patient would suffer from a composite of death from cardiovascular causes, non–procedure-related bleeding, stroke, or myocardial infarction.

## Discussion

The present work was aimed at evaluating the appropriateness of NI margins employed in trials comparing antithrombotic therapies in subjects undergoing TAVR. The present results showed that the studies generally have wide NI margins.

To provide a rough figure from a public health perspective, considering that the TAVR volume in the US was 72,991 in 2019 (9), the adoption of the antithrombotic therapies listed in this review, if NI would be eventually proven, could result in an additional 800–3,500 patients with unfavorable outcomes requiring medical management. Such wide NI margins are often adopted in study design to limit the big sample sizes that are usually required in NI studies in cardiovascular research.

Clearly, the definition of “wide” depends on the context, specifically on the clinical problem and the type of endpoint, especially in cases like this where reference is made to clinical hard endpoints. As an example, a “wider margin” is admitted when the primary endpoint does not include irreversible outcomes and the test intervention presents some advantages over the standard therapies, such as better tolerability or less adverse effects (10).

Regulatory authority recommendations for NI trials generally provide guidance on the methodological approach for margin definition, typically without delving into specific clinical contexts except for illustrative purposes. According to FDA guidelines (10), the fixed-margin method is generally recommended for defining the NI margin. This involves pre-specifying the NI margin. The NI is recommended to be defined as a value smaller than the entire effect of the control drug, usually based on a conservative estimate from previous studies, preferably placebo-controlled trials, to ensure the preservation of a clinically significant portion of the control's effect. Such an approach would ensure that the selection of the margin is a result of both statistical reasoning and clinical judgment. However, margin selection often seems to be influenced more by arbitrary clinical decisions and the availability of resources (11). This is evidenced by a review (12) in this field, which has revealed inadequate documentation of NI margin choice, particularly in studies published in journals with lower impact factors. Notably, the method was not reported in 158 out of 273 margins identified in the review. For the 115 margins where it was reported, only 40% defined the NI margin based on historical data about the comparator. This review highlights the fact that published studies do not seem to be fully compliant with existing recommendations. This underscores the need for stricter monitoring of NI studies, particularly concerning the justification and size of the margin, also when they are conducted beyond the scope of authorization processes, involving authors, journal reviewers, and ethics committees.

## Conclusions

The results of the present review pose serious concerns from ethical, clinical, and public health perspectives. Under such a scenario, if eventually NI will be claimed, the actual adoption of those antithrombotic therapies in patients undergoing TAVR could be likely unacceptable.
