# Supplementary material for: Non-inferiority designs in clinical trials for antithrombotic therapy in TAVR patients: did we go too far away by cutting corners?
Source: Front Cardiovasc Med. 2024 Jan 18;11:1327904. doi: 10.3389/fcvm.2024.1327904 (PMC10835793; doi:10.3389/fcvm.2024.1327904)

Table S1. Search Strings

|  | **PubMed** | 2/2/2023 |
| --- | --- | --- |
| 1 | ("anticoagulants"[mesh] OR “anticoagulants” OR “anticoagulant” OR “anticoagulation” OR “indirect thrombin inhibitors” OR “indirect thrombin inhibitor” OR "Platelet Aggregation Inhibitors"[mesh] OR "platelet aggregation inhibitors" OR "platelet aggregation inhibitor" OR “aggregation inhibitor” OR “aggregation inhibitors” OR antiplatelet OR antiplatelets OR antiaggregant OR antiaggregants OR “platelet antagonists” OR “platelet antagonist” OR antithrombic OR antithrombotic | 456434 |
| 2 | TAVI OR TAVR OR "transcatheter aortic valve replacement"[mesh] OR "transcatheter aortic valve replacement" OR "transcatheter aortic valve implantation" | 16806 |
| 3 | 1 AND 2 | 782 |
|  | **EMBASE** |  |
| 1 | exp anticoagulant agent/ OR anticoagulant agent.mp. OR anticoagulant agents.mp. OR anticoagulants.mp. OR anticoagulant.mp. OR anticoagulation.mp. OR Indirect Thrombin Inhibitors.mp. OR Indirect Thrombin Inhibitor.mp. OR Platelet Aggregation Inhibitors.mp. OR Platelet Aggregation Inhibitor.mp. OR Aggregation Inhibitor.mp. OR Aggregation Inhibitors.mp. OR antiplatelet.mp. OR antiplatelets.mp. OR Antiaggregant.mp. OR Antiaggregants.mp. OR Platelet Antagonists.mp. OR Platelet Antagonist.mp. OR Antithrombic.mp. OR antithrombotic.mp. OR exp antithrombocytic agent/ OR antithrombocytic agent.mp. OR antithrombocytic agents.mp. | 836248 |
| 2 | exp transcatheter aortic valve implantation/ OR transcatheter aortic valve implantation.mp. OR TAVI.mp. OR TAVR.mp. OR transcatheter aortic valve replacement.mp. | 33883 |
| 3 | 1 AND 2 | 3255 |
|  | **CENTRAL** |  |
| 1 | exp Anticoagulants/ OR anticoagulants.mp. OR anticoagulant.mp. OR anticoagulation.mp. OR indirect thrombin inhibitors.mp. OR indirect thrombin inhibitor.mp. OR exp Platelet Aggregation Inhibitors/ OR platelet aggregation inhibitors.mp. OR platelet aggregation inhibitor.mp. OR aggregation inhibitor.mp. OR aggregation inhibitors.mp. OR antiplatelet.mp. OR antiplatelets.mp. OR antiaggregant.mp. OR antiaggregants.mp. OR platelet antagonists.mp. OR platelet antagonist.mp. OR antithrombic.mp. OR antithrombotic.mp. | 40526 |
| 2 | TAVI.mp. OR TAVR.mp. OR exp Transcatheter Aortic Valve Replacement/ OR transcatheter aortic valve replacement.mp. OR transcatheter aortic valve implantation.mp. | 1214 |
| 3 | 1 AND 2 | 169 |

Figure S1. PRISMA flowchart


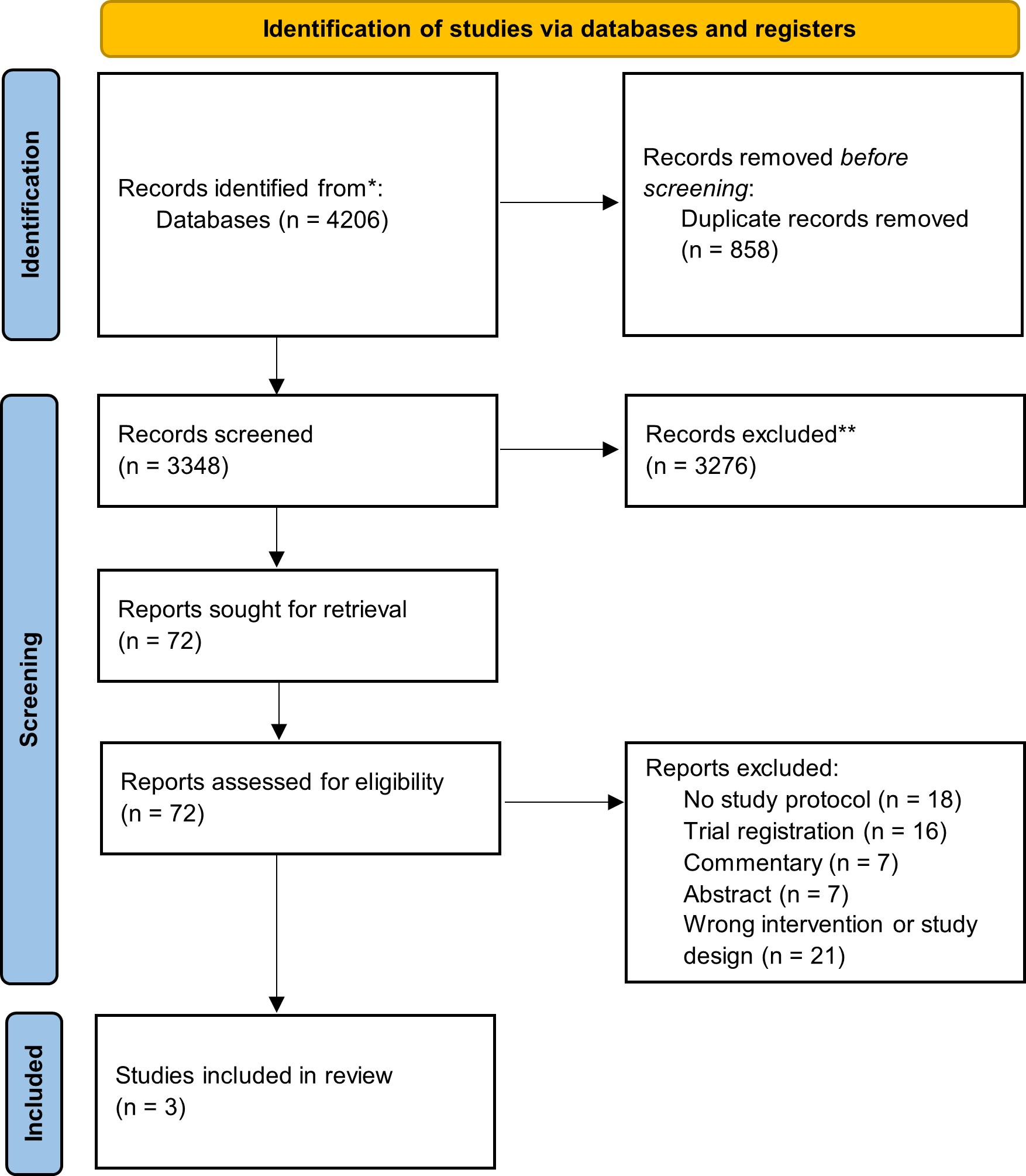

Supplement: Supplementary file 1 [file Table1.docx]
